# Primary adrenal lymphoma presenting as neurolymphomatosis: A case report

**DOI:** 10.1097/MD.0000000000037558

**Published:** 2024-03-22

**Authors:** Zhong Chen, Liyan Lin, Liqun Xu, Renhe Lin, Xiaoling Jiang

**Affiliations:** a Department of Neurology, Fuzhou Second Hospital, School of Clinical Medicine, Fujian Medical University, Fuzhou, P.R. China; b Department of Pathology, Clinical Oncology School of Fujian Medical University, Fujian Cancer Hospital, Fuzhou, P.R. China.

**Keywords:** neurolymphomatosis, peripheral nervous system, primary adrenal lymphoma

## Abstract

**Rationale:**

Primary adrenal lymphoma (PAL) is a very rare and highly aggressive disease. Neurolymphomatosis (NL) is a rare manifestation of lymphoma characterized by the infiltration of lymphoma cells into peripheral nerves, resulting in neurological symptoms. To date, there have been very few reported cases of PAL with NL. By reviewing the entire treatment process of the patient, we aim to enhance recognition of PAL complicated with NL and guide clinicians to pay attention to the diagnosis of such diseases. Early recognition and diagnosis of NL are crucial for appropriate management and treatment decisions.

**Patient concerns:**

We report a case of PAL in a 64-year-old female whose initial symptoms were pain and weakness in the left leg, which progressively worsened. In the half month before admission, the patient also showed signs of cranial nerve damage, such as diplopia and facial asymmetry.

**Diagnosis:**

Computed tomography of the abdomen revealed an occupying lesion in the left adrenal region. Electromyography and somatosensory evoked potential examination of the extremities suggested left lumbar plexus damage and complete damage to the right facial nerve. Adrenal biopsy confirmed diffuse large B-cell lymphoma.

**Interventions:**

The patient was treated with the R-CHOP scheme (rituximab, cyclophosphamide, doxorubicin, vincristine, and prednisone) combined with lenalidomide.

**Outcome:**

After 6 rounds of chemotherapy, the symptoms improved slightly. However, the condition progressed, and the patient passed away 1 year later.

**Lessons:**

Due to the nonspecific clinical presentation, patients with neurological damage should be alerted to the possibility of PAL and need to be evaluated thoroughly.

## 1. Introduction

Primary adrenal lymphoma (PAL) is extremely rare in clinical practice, and currently, there are fewer than 200 reported cases worldwide,^[1]^ accounting for less than 1% of extranodal lymphomas.^[2]^ The majority of PAL cases are non-Hodgkin lymphoma.^[3]^ When non-Hodgkin lymphoma invades the nervous system and causes damage to cranial nerves, nerve plexuses, nerve roots, and peripheral nerves, it is called neurolymphomatosis (NL).^[4]^ To date, there have been very few reported cases of PAL with NL.

In this study, we summarize the clinical data and treatment of one such case, in which a 64-year-old female patient with PAL developed symptoms of NL, such as facial nerve, oculomotor nerve, and lumbar plexus nerve damage.

## 2. Case presentation

The patient was a 64-year-old female who was admitted to the hospital because of left leg pain with weakness for 5 months, which worsened and was accompanied by double vision and facial asymmetry for 19 days. Physical examination showed clear consciousness, a poor mental state, a drooping right eyelid, a left pupil size of 2.5 mm and a right pupil size of 4.0 mm, reduced direct light reflex on the right side, diplopia (+), limited inwards and upwards movement of the right eye, disappearance of the right forehead wrinkle, a shallow right nasolabial groove, left rectus femoris muscle strength of grade 3, left tibialis anterior muscle strength of grade 1, and normal muscle strength in the other limbs. Muscle biopsy and nerve conduction studies suggested lumbar plexus damage on the left side and possible root damage on the right side.

**Figure 1.**
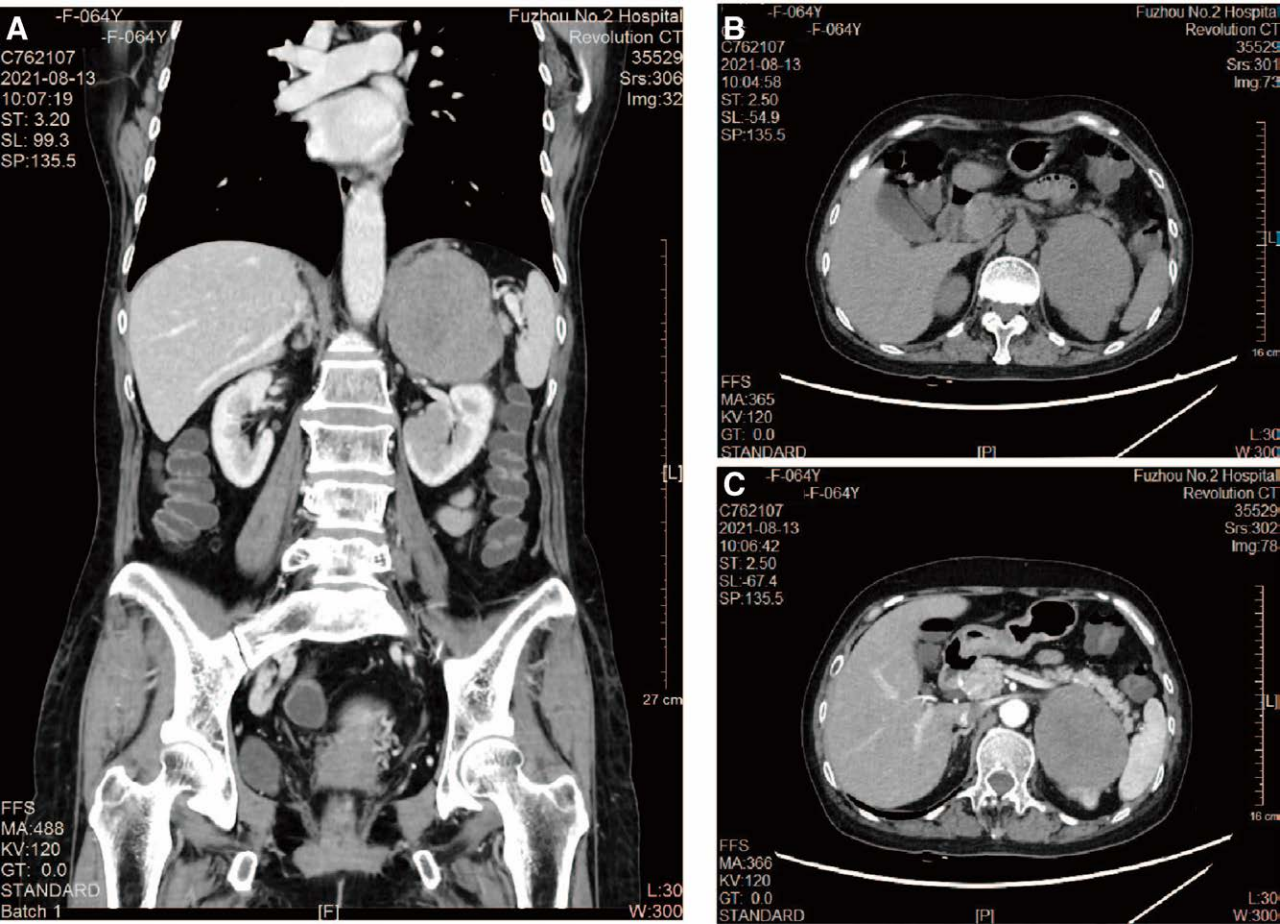
Computed tomography of the whole abdomen. A. Coronal enhancement; B. Cross-sectional plain scan; C. Cross-sectional enhancement.

Laboratory examination showed elevated lactate dehydrogenase levels (308 U/L). Routine blood tests, liver and kidney function, thyroid function, folic acid, vitamin B12, antinuclear antibodies, coagulation function, 8 infectious diseases, rheumatoid immune indicators, tumor markers, anti-neutrophil cytoplasmic antibodies, ganglioside antibodies, and paraneoplastic syndrome autoantibody spectrum were all within normal limits. Cerebrospinal fluid routine biochemical and paraneoplastic syndrome autoantibody spectrum tests were normal, and immature cells were not detected in the concentrated cerebrospinal fluid sample. Computed tomography (CT) showed a space-occupying lesion in the left adrenal region, mass size 8.0 × 6.7 × 7.8 cm; accessory spleen (Fig. 1). Positron emission computed tomography (PET-CT) revealed left adrenal infiltration and slightly metabolically inactive retroperitoneal lymph nodes, with increased metabolic activity in the upper palate and tonsils, a mildly increased metabolic nodule in the right lobe of the thyroid gland, and calcifications in the left lower lobe of the lung.

**Figure 2.**
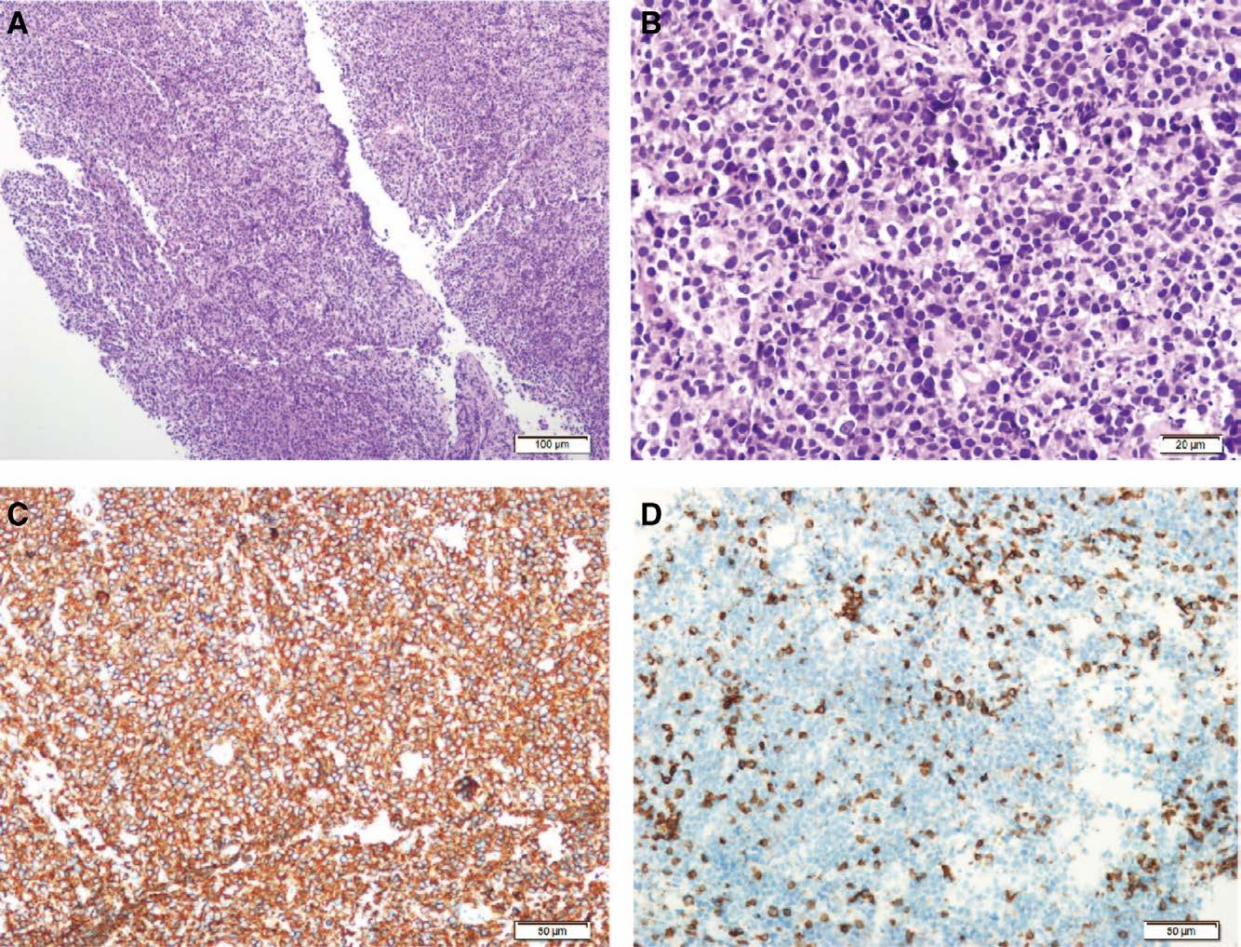
Haematoxylin eosin staining (HE) and immunohistochemical images of the left adrenal mass. A. Tumour cells were seen to be diffusely distributed (10×); B. Tumour cells were seen with medium-large size, abundant cytoplasm, basophilic, and deep nuclear chromatin (40×); C. CD20 staining showed diffuse positive tumor cells (20×); D. CD3 staining showed negative tumor cells with scattered positive T cells visible in the background (20×).

Pathological examination of the left adrenal mass biopsy revealed diffuse infiltration of medium-sized lymphoid cells, with basophilic cytoplasm and round, oval, or irregular nuclei with deep staining (Fig. 2). Based on the immunohistochemistry results, the patient was diagnosed with aggressive B-cell non-Hodgkin lymphoma (double expression of C-myc and Bcl-2 proteins). Immunohistochemistry results showed CD3 (−), CD20 (+), CD56 (−), Syn (−), Ki67 (+, 95%), CK (−), S−100 (−), CD21 (−), CD5 (−), CD43 (−), PAX−5 (+), CD30 (−), Bc1−2 (+, 100%), Bc1−6(+), CD10 (−), MUM−1 (+), C−myc (+, 80%), CD15 (−), CyclinD1 (−), and P53 (+, 100%). In situ hybridization results were negative for Epstein–Barr virus-encoded RNA. Fluorescence in situ hybridization (FISH) test results showed negative MYC isolation, negative BCL2 isolation, and positive BCL6 isolation.

After the diagnosis, the patient was transferred to the hematology department and received the rituximab, cyclophosphamide, doxorubicin, vincristine, prednisolone (R-CHOP) scheme combined with lenalidomide. After 6 courses of chemotherapy, the patient’s symptoms of double vision and lower limb weakness showed some improvement. Follow-up imaging revealed a reduction in the size of the left adrenal mass, measuring approximately 2.6 × 1.2 cm, compared to previous scans. However, due to the impact of the COVID-19 pandemic, the patient did not visit the hospital for her regular follow-up appointments. Five months later, the patient was admitted to a local hospital due to sudden confusion. Unfortunately, the patient passed away a few days later, and the specific cause of death is unknown.

## 3. Discussion

PAL is an extremely rare disease with high invasiveness. It often affects both adrenal glands and is more common in elderly males, with an onset age of 48 to 68 years old and a male to female ratio of 7:1.^[5,6]^ The patient in this case was an elderly female, and she showed unilateral adrenal involvement, which is even rarer. PAL lacks specificity in clinical symptoms, mainly including uncontrollable fever, fatigue, weight loss, abdominal pain, back pain, or skin pigmentation caused by adrenal insufficiency.^[7]^ However, due to the rich functional reserve of the adrenal glands, 90% of the adrenal parenchyma needs to be destroyed to cause adrenocortical insufficiency, so the adrenocortical function of some PAL patients can still be normal at the time of diagnosis.^[8]^

In terms of diagnosis, imaging examinations such as ultrasound, CT, magnetic resonance imaging (MRI) and functional imaging can be used as aids. CT shows bilateral adrenal involvement with heterogeneous density and homogeneous density, often with hemorrhage and necrosis. On T1-weighted MRI images, lesions show low signal intensity, while on T2-weighted images, they show high signal intensity and occasionally mixed signal areas.^[9,10]^ However, PAL still lacks characteristic changes on CT and MRI and is easily misdiagnosed as cortical adenoma, pheochromocytoma, metastatic carcinoma, etc. CT or ultrasound-guided percutaneous fine-needle aspiration biopsy pathology is the “gold standard” for the diagnosis of PAL.^[11]^ In the diagnosis of PAL, there are rarely other sites affected outside the adrenal gland, but during disease development, it may show a tendency to extensively involve multiple extranodal regions (such as the nervous system, liver, stomach).^[12]^ When lymphoma invades the nervous system, it is called neurolymphomatosis (NL), which is more difficult to diagnose clinically, and the number of cases reported is extremely small.

There are no accepted diagnostic criteria for NL, and confirmation of diagnosis requires nerve biopsy or autopsy. Some scholars have summarized the following diagnostic points: signs and symptoms of chronic peripheral nerve involvement; pathological examination confirms that the patient has lymphoma or leukaemia; MRI reveals corresponding nerve thickening and enhancement, and PET-CT examination suggests increased uptake of the corresponding nerve tracer; biopsy confirms lymphoma cell infiltration in the nerve bundle and its surrounding structures; and exclusion of other possible diseases. Since nerve biopsies are rarely performed and some inflammatory or infectious diseases can cause nerve thickening, the clinical diagnosis can be made as long as (1) (2) and (5) are met.^[13]^

In this case, the patient’s first symptom was pain and weakness in the left lower limb. The symptoms progressively worsened, and half a month before admission, she gradually developed double vision and facial paralysis. Physical examination revealed damage to multiple cranial nerves, including the facial nerve and oculomotor nerve. Abdominal CT scan and contrast-enhanced imaging suggested a left adrenal mass lesion, and percutaneous needle biopsy of the adrenal mass lesion confirmed diffuse large B-cell lymphoma (with dual expression of C-myc and Bcl-2 proteins). A complete PET-CT examination only showed infiltration of the left adrenal gland, ruling out the possibility of lymphoma involving lymph nodes or other organs. Therefore, the diagnosis was PAL. The patient also had progressive pain, weakness, and muscle atrophy in the left lower limb, and electromyography (EMG) showed peripheral nervous system damage involving the left lumbar plexus and right facial nerve, among others. EMG suggested positive sharp waves and fibrillation potentials in the left rectus femoris and left tibialis anterior muscles during relaxation, and during mild contraction, there were broad motor unit potentials and polyphasic waves in the left rectus femoris muscle, as well as absent motor unit potentials and polyphasic waves in the left tibialis anterior muscle. The left rectus femoris and right orbicularis oris muscles showed mixed-phase motor unit potentials. In summary, the EMG suggested left L3 and L4 paraspinal muscle damage, as well as root damage. It was recommended to conduct a complete lumbar plexus MRI examination, but the patient refused due to economic reasons. In summary, PAL is considered to involve the nervous system during disease progression and is consistent with the clinical diagnosis of NL.

The patient gradually developed symptoms of facial paralysis and other cranial nerve damage after 5 months of left lower limb weakness, which is consistent with the related report that NL is most commonly associated with painful polyneuropathy or radiculopathy, followed by cranial neuropathy,^[14]^ but the cranial MRI-enhanced examination of this case did not show obvious signs of nerve enhancement and thickening, which may be due to the following reasons: The lymphoma may have infiltrated the nervous system, such as the facial nerve and the arterio-ocular nerve, but it has not yet been visualized on imaging, which is similar to the report of Liu Shan et al,^[15]^ who reported that the lymphoma infiltrated the glossopharyngeal nerve and the vagus nerve without visualization on imaging. Due to some patients being immunocompromised, some lymphomas of the nervous system may not be abnormally enhanced on MRI, so it is recommended to repeat the enhancement MRI or PET-CT examination of the affected nerves or plexuses.^[16]^ Meanwhile, some studies have reported that LDH can be used as a tumor marker for PAL,^[5]^ and this patient’s LDH was significantly elevated, which is consistent with the literature.

Currently, there is no standard treatment for neurolymphomatosis, but as the primary lesion in this patient was PAL, treatment was focused on PAL. Clinical diagnosis and treatment guidelines recommend the R-CHOP scheme (rituximab, cyclophosphamide, doxorubicin, vincristine, and prednisone) as the standard first-line treatment for PAL.^[17]^ However, one-third of patients still suffer treatment failure or recurrence. To date, the most common pathological type of PAL is nongerminal center diffuse large B-cell lymphoma, accounting for more than 70% of cases.^[18]^ The latest research results suggest that for initial treatment, elderly patients, and patients with high-risk diffuse large B-cell lymphoma, the R-CHOP scheme combined with lenalidomide is superior to the R-CHOP scheme alone.^[19]^ Considering the patient’s pathological type, after being transferred to the hematology department, the patient received the R-CHOP scheme combined with lenalidomide. Unfortunately, the patient died a year later.

Some limitations in this case report are as follows. Firstly, it is a single rare case, and the findings have limited generalizability and statistical power. Secondly, the detailed pathological examination and immunohistochemistry helped reach the diagnosis, but a nerve biopsy, considered the gold standard, was not performed due to its invasiveness. MRI examination of affected nerves did not clearly show enhancement, possibly due to early stage infiltration or the patient’s immunocompromised status. Repeated advanced imaging may provide more insights. Finally, the follow-up information of the patient was limited. Long-term follow-up is essential to evaluate the efficacy of the treatment and assess the patient’s outcomes.

## 4. Conclusions

In summary, PAL has a very low incidence rate, and its manifestation as NL is even rarer. Due to the lack of specific clinical symptoms, physicians should be aware of the possibility of PAL in patients with nervous system damage and conduct a comprehensive evaluation. Early diagnosis and treatment are closely related to the patient’s quality of life and prognosis. Therefore, neurologists and hematologists should increase their understanding of this disease, strengthen interdisciplinary cooperation, and better guide clinical work.

## Acknowledgments

We gratefully acknowledge the patient for her participation and consent.

## Author contributions

**Data curation:** Liyan Lin, Liqun Xu.

**Investigation:** Renhe Lin.

**Writing – original draft:** Zhong Chen.

**Writing – review & editing:** Zhong Chen, Xiaoling Jiang.
